# PB.01: Second-look ultrasound examination of the breast following MRI: MR and sonographic findings

**DOI:** 10.1186/bcr3503

**Published:** 2013-11-08

**Authors:** K Alrawi, J Bansal, P Young

**Affiliations:** 1 Cardiff & Vale Breast Centre, University Hospital Llandough, Cardiff & Vale University Health Board, Cardiff, UK

## Introduction

MRI is widely accepted to be the most sensitive imaging modality for detecting breast cancer, but has relatively low specificity. Often additional enhancing areas are identified on MRI that require further investigation. In this study we evaluated second-look ultrasound following breast MRI and the impact of this on patient management.

## Methods

A retrospective review was undertaken of all breast MRIs performed between July 2010 and December 2012. Patients who had further evaluation with second-look ultrasound were reviewed. Clinic letters were also reviewed to identify any subsequent change in patient management.

## Results

A total of 261 breast MRI scans were performed over this time period. Fifty-two (19.9%) had a second-look ultrasound performed; 24 for mass-like lesions and 28 for nonmass-like lesions identified on MRI. In total, 18/24 (75%) mass-like lesions had a corresponding ultrasound abnormality, and 12/28 (42.8%) nonmass-like lesions had a corresponding ultrasound abnormality. Thirty biopsies were performed (57.6%) and of these nine (17.3%) were malignant. Malignant lesions were equally distributed between mass-like and nonmass-like lesions. In total, 7/9 malignant lesions were scored U4 or U5, demonstrating features suspicious of malignancy. Management was altered in all nine cases.

## Conclusion

MR-directed second-look ultrasound is a valuable tool in diagnostic work-up. Mass-like lesions on MR are more likely to have an ultrasound correlate. Most malignant lesions had clearly malignant features on ultrasound.

